# Clinical radiological aspects of primary endodontic lesions with secondary periodontal involvement

**Published:** 2017

**Authors:** R Jivoinovici, Ileana Suciu, I Gheorghiu, Ioana Suciu

**Affiliations:** * Faculty of Dental Medicine, “Carol Davila” University of Medicine of Pharmacy, Bucharest, Romania

**Keywords:** endo-periodontal lesion, calcium hydroxide, chlorhexidine

## Abstract

Damage of pulp tissue usually begins in the coronal pulp. Its mistreatment or its lack of on time detection determines the progressive inclusion of the whole endodontic space in its evolution, opening the way of its expansion in the surrounding tissues of the tooth, and on the marginal apical tissue.

**Aim.** The goal of this study was to highlight that the primary endodontic lesions with secondary periodontal implication healed and bone repair was obtained due to a proper disinfection and an adequate sealing of the endodontic system.

In primary endodontic lesion with secondary periodontal involvement, endodontic treatment is required in the first stage followed by specific periodontal treatment. The prognosis is good if an appropriate endodontic approach is chosen, depending on the stage of the periodontal disease and the treatment response. The identification of the etiological factors is the most important to establish the appropriate treatment.

In all clinical cases selected in this article, the healing tendency was noticed after an adequate disinfection and sealing of the endodontic system.

## Introduction

Typically, pulp and periodontal pathology respectively, has an evolution of its own. However, there are clinical situations in which the two types of tissue lesions events intertwine, this context being generically known as an endo-periodontal syndrome.

Consistent with the analysis of Blomlof [**1**], in a retrospective study, the endodontic infection induces periodontal pocket evolvement, and thus the affection of the attachment, leading to the progression of periodontitis. Thus, a primary endodontic treatment is rather preferred as a stage, since an aggressive removal of the periodontal ligament and cementum, during the endodontic therapy, adversely affects the periodontal healing [**4**-**6**]. The extension of the periodontal inflammation in the pulp and vice versa is facilitated by the existence of the anatomic links between the two tissues, which allows the establishment of a reciprocal pathological influence.

Regarding the endo-periodontal anatomical pathways, they are represented by the apical foramen, side and accessory channels and dentinal tubules [**2**,**3**].

## Case report 1

Patient A.S., 38 years old, presented to be treated for pain and swelling on 2.2.

Subjective symptoms: egress feeling tooth 2.2, intense pain.

On examination: the presence of fistulas in the right vestibular fixed mucosa of tooth 2.2 was indicated, sensitivity to percussion in shaft, depth survey of 2.2 mezial 4 mm. Vitality tests: negative with dry ice.

**Fig. 1:**
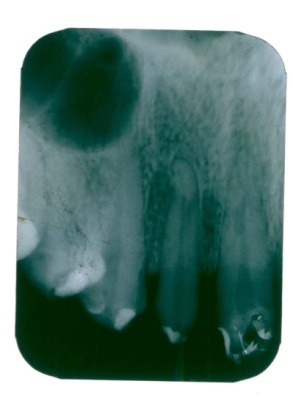
Periapical radiolucency and partial resorption of the interdental septum on 2.2 tooth

Radiographic examination: the initial radiological image (**Fig. 1**) presented a periodontal space widening of the 2.2 tooth with periapical and lateral radiolucency and triangulation on the interdental septum.

Diagnostic: exacerbations of chronic apical periodontitis, accompanied by alveolar bone loss marginally.

Endo-periodontal lesion with primary endodontic pathology and secondary periodontal involvement.

Treatment: first stage: endodontic treatment, which consisted of:

- debridement;

- investigating;

- medical dressing of Ca (OH)2 2 to 4 weeks;

- root canal length by using a lateral condensation technique.

An appropriate endodontic therapy was performed together with a periodontal treatment, which consisted of:

- gingival debridement by removing plaque, oral biofilm and their products;

- supragingival scaling;

- professional subgingival scaling;

- irrigation with chlorhexidine 0.2% (Dentaton, Ghimas Casalecchio di Reno spa, Bologna, Italy), followed by an instillation of chlorhexidine gel 1% (Chlorhexamed, GSK, Brenford, UK);

- patient education for acquiring a main sanitation system by brushing and by using secondary aids.

**Fig. 2:**
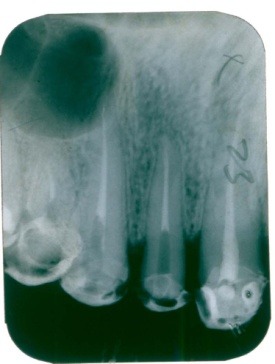
The restriction zone radiolucency - periapical and interdental bone recovery trend at 2.2

The subsequent assessment on the image (**Fig. 2**) to 12-months noted a canal obturation quality as well as a decrease until no radiolucent periapical and side.

**Case report 2**

Patient I.U., 58 years old, presented to treatment for tooth 3.4, with a history of multiple episodes of moderate intensity, painful tooth was contained within a fixed prosthetic work.

Subjective symptoms: pain of moderate intensity on 3.4.

On examination: Axial percussion, positive vitality tests negative.

**Fig. 3:**
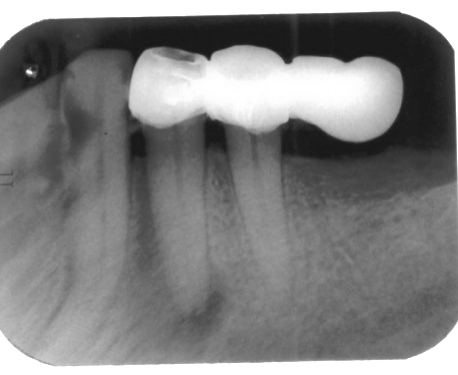
Tooth 34 with periapical contoured radiolucency

Radiographic examination: 3.4 with a periodontal space widening (**Fig. 3**) with periapical radiolucency, circumscribed and lateral, located on mesial and distal sides of the tooth, accompanied by widening desmodontal space.

Diagnostic: exacerbations of chronic apical periodontitis.

Endo-periodontal lesion with primary endodontic pathology and secondary periodontal involvement.

Treatment: in the first stage, the endodontic treatment consisted of:

- debridement;

- instrumentation;

- medical dressing of Ca (OH)2 2 to 3 weeks;

- obturation lasting channel by using the lateral condensation technique. 

An appropriate endodontic therapy was performed and periodontal treatment consisted of:

- supragingival scaling;

- professional subgingival scaling;

- irrigation with chlorhexidine 0.2% (Dentaton, Ghimas Casalecchio di Reno spa, Bologna, Italy), followed by an instillation of chlorhexidine gel 1% (Chlorhexamed, GSK, Brenford, UK);

- patient education for acquiring a main sanitation system by brushing and by using secondary aids.

**Fig. 4:**
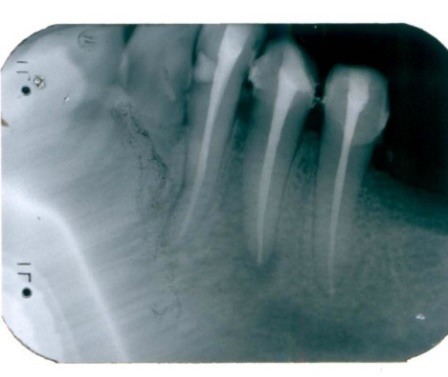
Tooth 3.4 reduced periapical radiolucency

Control radiography was performed at 6 months (**Fig. 4**), noting the quality, and reduced periapical radiolucency.

**Case report 3**

Patient I.O., 39 years old, accused intermittent episodes of pain in history, which started two years before and the presence of fistula in the right tooth 4.6, 4.7.

Subjective symptoms: the presence of fistula.

On examination: mucosa vestibular fistula fixed presence in the region of tooth 4.7, vitality tests negative, carried out with ice, ax percussion sensitivity, depth sounding the centro-vestibular site - 6 mm and the mesio-vestibular site - 5 mm.

**Fig. 5:**
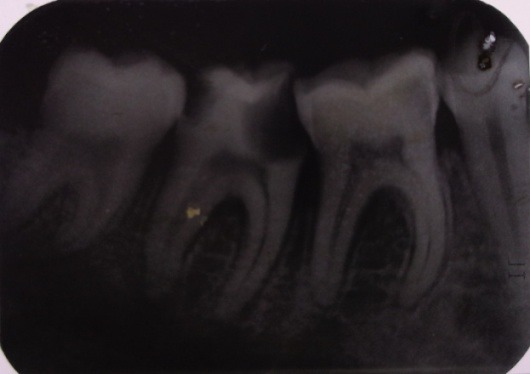
Well defined radiolucency circumscribing the apex of the tooth root mesial and distal 4.7 and radiolucency area at furcation with demineralization of interradicular septum

Radiology ic: initial radiograph (**Fig. 5**) had 4.7 teeth with periapical radiolucent mesial and distal to the roots, radiolucency at furcation and desmodontal space widening.

Diagnosis: Chronic apical periodontitis exacerbation.

Endo-periodontal lesion, primary endodontic pathology, and secondary periodontal involvement.

Treatment: endodontic first stage of treatment, which consisted of:

- debridement;

- instrumentation;

- medical dressing with Ca (OH)2 2 to 3 weeks;

- lasting obturation technique using lateral condensation. 

Afterwards, an appropriate endodontic therapy was performed and the periodontal treatment consisted of:

- gingival debridement by removing plaque, oral biofilm and their products;

- supragingival scaling;

- professional subgingival scaling;

- 2% chlorhexidine irrigation (Dentaton, Ghimas Casalecchio di Reno spa, Bologna, Italy), followed by an instillation of chlorhexidine gel 1% (Chlorhexamed, GSK, Brenford, UK);

- patient education for acquiring a main sanitation system by brushing and by using secondary aids.

**Fig. 6:**
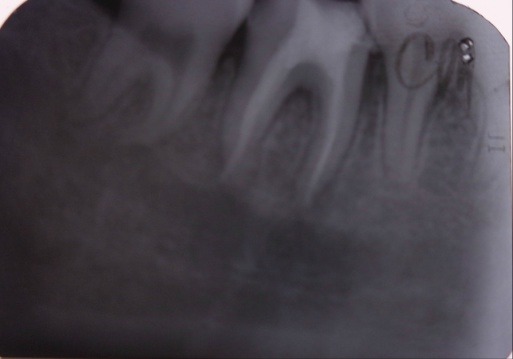
Tooth 4.7 with a decreased periapical and furcation radiolucency

On radiography control after one year (**Fig. 6**), there was a tendency of healing and periapical osteitis remineralization of the interdental septum with a radiolucent decrease in furcation, the evolution being considered favorable.

**Case report 4**

Patient I.V., 35 years old, presented for endodontic re-treatment in tooth 2.6.

Subjective symptoms: moderate mastication embarrassment at the level of 2.6.

On examination: sensitivity to percussion in the shaft.

**Fig. 7:**
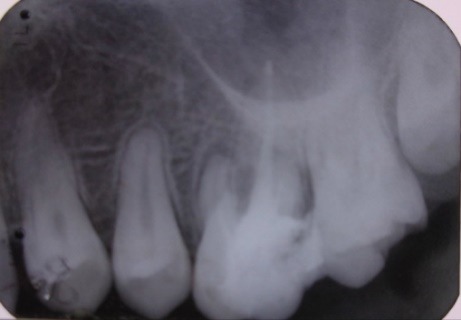
Tooth 2.2 with circumscribed radiolucency at the mesial root-level and incomplete root canal

Radiographic examination: tooth 2.6 (**Fig. 7**) with an incomplete root canal with periapical radiolucency at the mesial root and disto-vestibular and vestibular bone demineralization of furcation.

Diagnostic: 2.6 fibrous chronic apical periodontitis.

Endo-periodontal lesion pathology, endodontic primary and secondary periodontal involvement.

Treatment: in the first step, an endodontic retreatment was performed, which consisted of:

- debridement;

- instrumentation;

- medical dressing of Ca (OH) 2 for 3 weeks;

- lasting obturation technique using lateral condensation. 

Afterwards, an appropriate endodontic therapy and a periodontal treatment were performed, which consisted of:

- supragingival scaling;

- professional subgingival scaling;

- 2% chlorhexidine irrigation (Dentaton, Ghimas Casalecchio di Reno spa, Bologna, Italy), followed by an instillation of chlorhexidine gel 1% (Chlorhexamed, GSK, Brenford, UK);

- patient education for acquiring a main sanitation system by brushing and by using secondary aids.

**Fig. 8:**
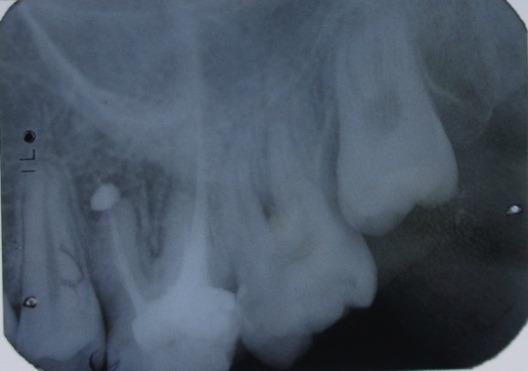
Periodontal space with periapical radiolucency at mesial and disto-vestibular root circumscribing apex -tooth 2.6

The radiograph verification (**Fig. 8**) of the root canal after 6 months was found in the roots radiolucency periapical and disto-mesial-vestibular level.

**Fig. 9:**
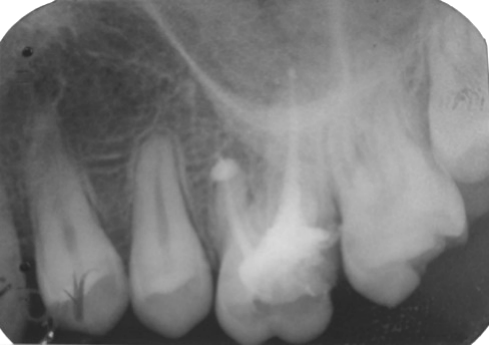
A periapical restriction radiolucent area on mesial, disto-vestibular and furcation level was observed on 2.6

The radiological image control (**Fig. 9**) was performed at 1 year after the treatment tended to show remineralization and reduction radiolucent of distal septum circumscribing apexes and disto-mesial buccal roots and the furcation in the presence of a moderate amount of excess sealant.

**Case report 5**

Patient C.M., 25 years old, presented for restoration of coronal tooth 1.1, 1.2.

Subjective symptoms: no complaints.

On examination: being aware of secondary caries in the teeth that approximated 1.1 and 1.2.

**Fig. 10:**
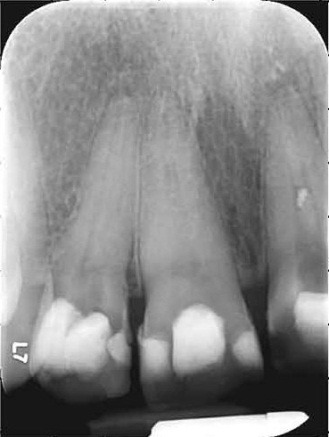
Presence of apical and lateral radiolucent to the level of 1.2

Radiographic examination: the presence of periapical radiolucent and demineralization on the mesial interdental septum was observed in the tooth 1.2 (**Fig. 10**).

Diagnosis: 1.1, 1.2 fibrous chronic apical periodontitis.

Endo-periodontal lesion, primary endodontic pathology, and secondary periodontal involvement.

Treatment: the first stage presupposed the performance of an endodontic treatment, which consisted of:

- debridement;

- instrumentation;

- medical dressing with Ca (OH)2 2 to 3 weeks;

- lasting obturation technique using lateral condensation. 

Afterwards, making an appropriate endodontic therapy and periodontal treatment was performed, which consisted of:

- supragingival scaling;

- professional subgingival scaling;

- irrigation with chlorhexidine 0.2% (Dentaton, Ghimas Casalecchio di Reno spa, Bologna, Italy), followed by an instillation of chlorhexidine gel 1% (Chlorhexamed, GSK, Brenford, UK);

- patient education for acquiring a main sanitation system by brushing and by using secondary aids.

**Fig. 11:**
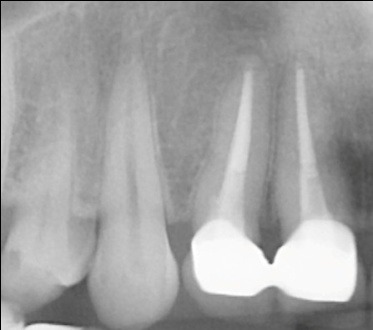
Teeth 1.1, 1.2 complete fillings of channels with reduced apical and lateral area radiolucency

Control radiography was performed at 1 year and a half after treatment (**Fig. 11**) and presented a decrease in the apical radiolucent from the tooth 1.2.

## Discussions

Endo-periodontal lesions can occur because of the vascular connection between the pulp and the periodontium; therefore, a proper disinfection and sealing of the endodontic system, might contribute to the healing of the primary perio-endo lesion [**8**,**9**]. The microbial germs and toxic products from infected root canal [**7**], crossing the channels and accessories from furcation, can promote fistula formation and affect the tooth supporting apparatus.

The endodontic infection increases the possibility of forming a periodontal pocket and is a risk factor in the progression of periodontal disease, so a primary endodontic injury that drains the periodontal ligaments should be initially treated with an endodontic treatment because the removal of aggressive periodontal ligaments and the cementum periodontal healing have a negative influence.

The symptoms of periodontitis marginal, such as the presence of purulent deep periodontal pockets, gingival swelling, tooth mobility, bone resorption radiologically demonstrated, could be as good as a cause triggered by an endodontic aspect.

Sometimes, though more rarely, a radiological image of periapical radiolucency may be the consequence of mortification and pulp infections induced by periodontitis marginal developments.

The correlation between the pulp and the periodontal pathology is not confined to the possibility of triggering the first of the second [**10**,**11**]. Pulpopathies occurring amid already installed can worsen the progress of periodontal diseases.

Regarding the periodontitis marginal effect on the dental pulp, the same anatomical pathways that govern the expansion of pulp pathology marginal periodontal structures provide the opposite effect and marginal periodontal pathology of the pulp body.

The pulpopathies role in causing periodontal pathology is controversial, lacking evidence of correlations between the clinical course of the disease stage periodontal lesion severity and the histopathological form or pulp.

The primary endodontic lesions usually heal after an endodontic disinfection and, the sealing of the system and the control radiography is performed after one year when bone repair can be observed; it requires an invasive periodontal therapy that should be postponed until after the completion of the endodontic treatment.

## Conclusions

The endo-periodontal syndrome, onset endodontic root canal treatment, promptly and rigorously leads to a regeneration of both injury osteitis, apical and marginal.

The primary endodontic secondary affection with poor periodontal must first be treated by endodontic therapy. The results of the treatment should be evaluated after six months and only then, the need to apply periodontal therapy should be considered. The prognosis depends on the severity of the periodontal damage, and the periodontal therapy applied to the patient’s response to treatment. Before the initiation of periodontal treatment, an intraductal placement of calcium hydroxide paste prop is indicated, renowned due to its antibacterial and anti-inflammatory inhibition of root resorption.

The combined lesion requires an endodontic treatment approach, but at the same time, a specific periodontal treatment is mandatory, in order to overcome the secondary periodontal affection.
